# Are gastric metastases of renal cell carcinoma really rare? A case report and systematic review of the literature

**DOI:** 10.1016/j.ijscr.2021.105867

**Published:** 2021-04-06

**Authors:** Thomas Prudhomme, Charlotte Maulat, Guillaume Péré, Fatima-Zohra Mokrane, Michel Soulié, Fabrice Muscari

**Affiliations:** a Department of Digestive Surgery, Toulouse University Hospital, 31059, Toulouse, France; b Department of Urology and Kidney Transplantation, Toulouse University Hospital, 31059, Toulouse, France; c Department of Radiology, Toulouse University Hospital, 31059, Toulouse, France

**Keywords:** Renal cell carcinoma, Gastric metastases, Surgical treatment, Systemic treatment, Metastatic recurrence, Case report

## Abstract

**Introduction:**

Renal cell carcinoma (RCC) represents above 3 % of all cancers. At diagnosis, above 25 % of patients with RCC present an advanced disease. Gastric metastasis of RCC is associated with poor outcome. We report the case of a patient treated for a gastric metastasis of RCC and we conducted a systematic review of the literature to report all published cases of RCC patients with gastric metastasis.

**Case presentation:**

In December 2010, a 61-year-old man was treated by open partial nephrectomy for a localized right clear cell RCC. In September 2018, a metachronous gastric metastasis was found on CT scan. The lesion was located on the lesser curvature of the stomach, measuring 4.5 cm long axis. No other secondary lesions were identified. A laparoscopic wedge resection, converted to laparotomy was performed. Two years later, in September 2020, a CT scan was performed, revealing a 17 mm adenopathy behind the hepatic hilum and a surgical management was performed, including a lymph node dissection of the hepatic hilum and the hepatic artery. Actually, he remains healthy.

**Clinical discussion and conclusion:**

Our systematic review suggests that solitary gastric metastasis of RCC are scarce. In comparison of patients with multiple metastatic sites, the median survival of patients with solitary gastric metastasis is longer.

## Highlights

• Solitary gastric metastasis of RCC are scarce.
• When feasible, surgical or endoscopic treatment of gastric metastasis should be performed.
• Risk of metastatic recurrence is significant and must be taken into consideration in the therapeutic strategy.

## 1 Introduction

Renal cell carcinoma (RCC) represents above 3 % of all cancers, with the highest incidence occurring in Western countries [1]. At diagnosis, above 25 % of patients with RCC present an advanced disease, including locally invasive or metastatic cancer [2,3]. The mean survival time of patient with metastatic RCC is approximately 13 months [1].

The most common metastatic RCC sites are the lung and bone (up to 60 % and 40 % of patients with metastases) [4]. However, unusual sites of metastasis are characteristics of RCC and any organ site can be involved, including the thyroid, pancreas, skeletal muscle and skin.

Gastric metastasis of RCC has been described [[5], [6], [7], [8], [9], [10], [11], [12], [13], [14], [15], [16], [17], [18], [19], [20], [21], [22], [23], [24], [25], [26], [27], [28], [29], [30], [31], [32], [33], [34], [35], [36], [37], [38], [39]] and it is associated with poor outcome, with only a third of patients being alive after one year [23]. Usually, gastric metastases are asymptomatic, single and located in the gastric body or fundus [23,33].

Thus, these metastases are diagnosed on routine CT scans as part of the monitoring of RCC after local treatment. Gastrointestinal bleeding and anemia are the most common presentations of symptomatic gastric metastasis [23,33].

We herein report the case of a patient, who underwent a wedge resection for a gastric metastasis of RCC, which is healthy at 2-years of follow-up. Furthermore, we conducted a systematic review of the literature to report all published cases of RCC patients with gastric metastasis.

## 2 Case report

In December 2010, a 61-year-old man was referred to our academic center for the management of a localized right clear cell RCC, without synchronous metastases. It was an upper polar lesion, measuring 2.5 cm long axis, essentially exophytic, posterior, entirely above the polar lines, without vascular contact, classified 5p RENAL score.

An open right partial nephrectomy was performed by an experienced surgeon and histological results highlighted a 2.5 cm clear cell RCC, Furhman grade III, classified pT3aN0R0 due to the extension to perinephric tissues, but not beyond Gerota Fascia.

According to EAU (European Association of Urology) guidelines [40], a surveillance has been validated including semi-annual monitoring by CT scan in the 1 st year, then annual monitoring by CT scan in the following 2 years and every 2 years thereafter.

In Mars 2012, a localized homolateral recurrence has been highlighted at CT scan. It was a lower polar lesion, measuring 4.3 cm long axis, essentially endophytic, anterior, close to the collecting system, with polar line crosses and venous contact, classified 9 ah RENAL score, Supplemental Fig. 1. There was no evidence of metastasis on this CT scan.

An open right radical nephrectomy was performed and histological examination reported a 3.5 cm clear cell RCC, Furhman grade III, classified pT3aN0R0 due to the extension to the renal sinus fat.

According to EAU guidelines [40], a surveillance has been validated.

In September 2018, a metachronous gastric metastasis was found on CT scan. The lesion was located on the lesser curvature of the stomach, measuring 4.5 cm long axis. No other secondary lesions were identified, Supplemental Fig. 2. The patient had no digestive symptoms, no hematemesis, no melena.

A surgical procedure has been validated: a laparoscopic wedge resection, converted to laparotomy has been performed.

Histological examination reported a proliferation of atypical cells containing round clear nuclei and irregularly shaped nuclei with prominent nucleoli using hematoxylin-eosin staining. Immunostaining revealed negativity for all epithelial markers (CK7, CK20) and positivity for proximal tubular epithelial markers (CD10, Vimentine). These characteristics led to the conclusion of gastric metastasis of a clear cell RCC with 30 % sarcomatoid contingent, with negative resection margins.

The patient had an uneventful postoperative course and was discharged on the 8th day.

Two years later, in September 2020, a CT scan was performed, revealing a 17 mm adenopathy behind the hepatic hilum and a surgical management was performed, by an experienced surgeon, including a lymph node dissection of the hepatic hilum and the hepatic artery. Histological examination reported a node metastasis of the clear cell RCC, in hepatic artery lymph node dissection. Actually, he remains healthy.

This case has been reported in line with the SCARE 2020 criteria [41].

## 3 Systematic review

Literature about gastric metastasis of RCC was realized using electronic database Medline via PubMed (1950–2018).

**Fig. 1:**
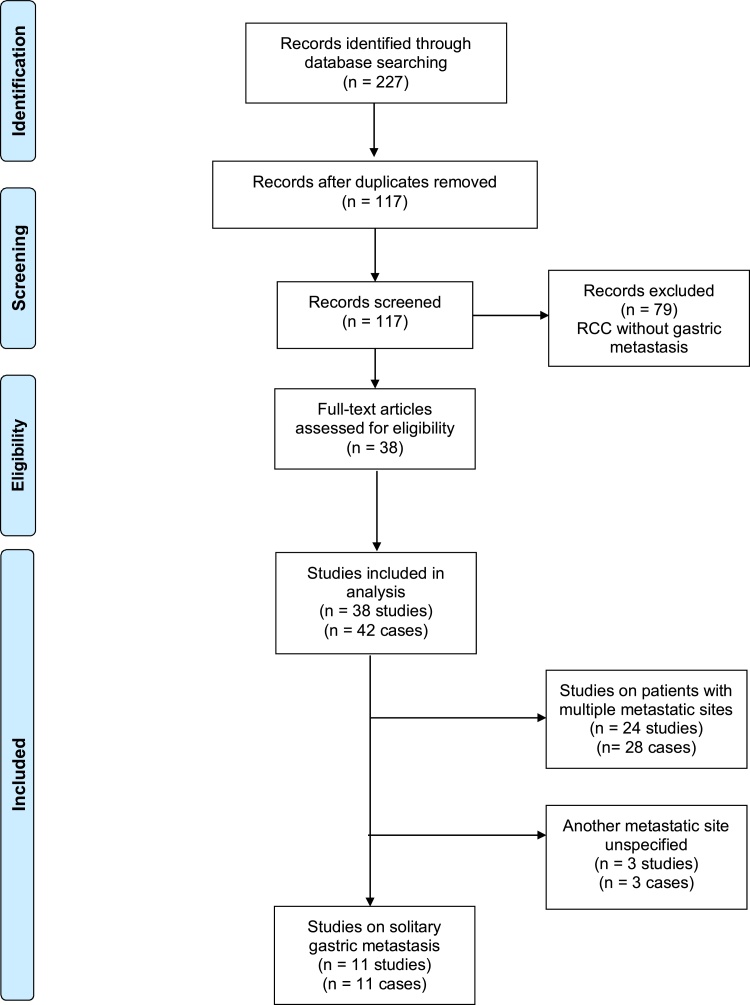
PRISMA flow diagram.

Key terms used included “renal cell carcinoma,” “stomach metastasis,” and “gastric metastasis” (Fig. 1). References in the identified articles were used to identify more relevant studies.

RCC with solitary gastric metastasis cases (n = 11) are summarized in Table 1.

**Table 1:** Summary of solitary gastric metastasis of RCC cases reported in the literature.

*RCC with Solitary Gastric Metastasis*							
*Author*	*Year*	*Age*	*Sex*	*Location*	*Interval (years)*	*Treatment*	*Outcomes*
Sullivan et al. [5]	1980	69	M	L	7	Antrectomy	*ND*
Odori et al. [12]	1998	59	M	U	4	Total gastrectomy	17-month survival
Picchio et al. [13]	2000	64	F	M	14	Subtotal gastrectomy	6-month survival
Saidi et al. [21]	2007	*ND*	*ND*	M	10	Partial gastrectomy	18-month survival
Sugasawa et al. [27]	2010	69	M	U	19	Partial gastrectomy	12-month survival
Namikawa et al. [29]	2012	65	M	U	23	Partial gastrectomy	2-month survival after therapy
Kim et al. [36]	2012	79	M	M	0	Endoscopic treatment	6-month survival after therapy
Kumcu et al. [32]	2014	59	M	M	4	Partial gastrectomy	*ND*
Rita et al. [33]	2014	77	M	M	2	Endoscopic treatment	3-month survival after therapy
Akay et al. [34]	2016	72	M	U	20	Chemotherapy	*ND*
Abu Ghanimeh et al. [38]	2017	67	M	L	3	Endoscopic treatment	1-year survival
Prudhomme et al.	2021	69	M	M	6	Wedge resection	26 months survival

Location (L: Lower body of the stomach; M: Middle body of the stomach; U: Upper body of the stomach).

The median patients age (IQR) was 68.0 (62.8–73.3) years, of whom 82 % were men. The most frequent locations of metastasis were the middle body of the stomach (45 %), followed by the upper body of the stomach (36 %) and the lower body of the stomach (18 %). The median time (IQR) between the diagnosis of RCC and metachronous gastric metastasis was 8.5 (3.8–19.3) years. 1 patient presented RCC with synchronous gastric metastasis. A surgical treatment of the metastasis was performed in 64 % of the patients and an endoscopic treatment in 27 % of the cases. Endoscopic treatment was a submucosal resection without positive surgical margins. 1 patient received a palliative chemotherapy. Median patient survival (IQR), after treatment, was 9 months (3.4–15.8).

RCC with multiple or unspecified metastatic sites (including stomach) cases (n = 31) are summarized in Table 2.

**Table 2:** Summary of RCC with gastric and multiple or unspecified metastasis site cases reported in the literature.

*RCC with Multiple or Unspecified Metastasis Site (including Gastric)*								
*Author*	*Year*	*Age*	*Sex*	*Location*	*Interval (years)*	*Treatment*	*Additional metastases*	*Outcomes*
Ibanez Olcoz et al. [6]	1989	60	F	M	1.8	Palliative treatment	Lung, brain	Died 33 days after operation
Marquez et al. [7]	1992	70	M	M	0.1	Palliative treatment	Pleura	Died 4 weeks
Durous et al. [8]	1992	66	M	U	12	Interferon	Lung, adrenal	*ND*
						No surgical treatment		
Herrera Puerto et al. [9]	1993	63	M	L	0.1	Palliative treatment	Lung	Died 4 weeks after nephrectomy
Boruchowicz et al 10	1995	48	M	U	1	Chemotherapy	Lung, liver, esophagus	Died 4 months after therapy
Blake et al. [11]	1995	63	M	M	6	Palliative embolization	Lung	No complications at 5 months
Mascarenhas et al. [14]	2001	66	M	U	7	Partial gastrectomy	Lung, pleura	3-year survival
Kok Wee et al. [15]	2004	60	M	M	20	*ND*	*ND*	*ND*
Kobayashi et al. [16]	2004	68	M	L	11	Total gastrectomy	Lung, liver, pancreas	Died at 2 years
						Chemotherapy		
Suarez Fonseca et al. [17]	2004	61	F	M	4	Palliative treatment	Lung	6-month survival
Lamb et al. [18]	2005	69	F	M	3	Palliative embolization	Lung, thyroid	Died 23 month after therapy
Riviello et al. [19]	2006	68	M	U	11	Total gastrectomy	Lung, brain, pancreas	Died 24 month after therapy
Portanova et al. [20]	2006	67	F	M	5	Total gastrectomy	Pancreas	*ND*
Pezzoli et al. [22]	2007	78	M	U	5	Endoscopic treatment	*ND*	Died 6 months after therapy
Pollheimer et al. [23]	2008	69	M	M	4	Tamoxifen	Lung, bone, adrenal	Died 19 months after therapy
						No surgical treatment		
Pollheimer et al. [23]	2008	77	M	L	6	Interferon	Lung, bone	Died 4 months after therapy
						No surgical treatment		
Pollheimer et al. [23]	2008	83	F	L	2	Interferon	Lung, liver, pancreas	Died 5 months after therapy
						No surgical treatment		
Pollheimer et al. [23]	2008	65	F	*ND*	13	Partial gastrectomy	Lung, brain	Died 3 months after therapy
Pollheimer et al. [23]	2008	69	M	M	9	Sunitinib	Lung, bone	2-year survival
						Partial gastrectomy		
Yamamoto et al. [24]	2009	74	M	M	5	Partial gastrectomy	Brain	Died 1 month after therapy
Kibria et al. [25]	2009	53	M	U	0	Palliative treatment	Lung, bone	Died 2 months after therapy
Maeda et al. [26]	2009	49	M	M	2	Partial gastrectomy	Lung	Died 15 months after therapy
Tiwari et al. [28]	2010	58	F	L	0	Subtotal gastrectomy	Lung	Died 2 months after therapy
Eslick et al. [39]	2010	65	M	M	9	Endoscopic treatment	Bone	6-year survival
Sakurai et al. [30]	2014	61	M	M	2	Partial gastrectomy	Lung, bone, brain	Died 4 months after therapy
Ikari et al. [31]	2014	64	M	M	12	Endoscopic treatment	Pancreas	30-month survival after therapy
Sogabe et al. [35]	2016	53	M	L	2	Sunitinib	Mediastinal Lymph Node	4-year survival after therapy
Arakawa et al. [37]	2018	80	F	M	0	Axitinib	Lung, liver	*ND*
						No surgical treatment		
Kinoshita et al. [47]	2019	60	M	M	3	Partial gastrectomy	Gallbladder	9-months survival
Bernshteyn et al. [48]	2019	68	M	M	*ND*	Endoscopic treatment	*ND*	*ND*
Orosz et al. [49]	2020	73	M	U	*ND*	Endoscopic treatment	Brain	*ND*

Location (L: Lower body of the stomach; M: Middle body of the stomach; U: Upper body of the stomach).

At RCC gastric metastasis diagnosis, 73 % of the patients presented multiple metastatic sites, mainly lungs and bones. The median patients age (IQR) was 66.0 (60.0–69.0) years, of whom 74 % were men. The median time (IQR) between the diagnosis of RCC and metachronous gastric metastasis was 5.0 (2.0–9.5) years. 3 patients presented RCC with synchronous gastric metastasis. 35 % of the patients had a surgical treatment, 16 % an endoscopic treatment, 23 % a systemic therapy and 26 % a palliative treatment. Endoscopic treatment was mucosal and submucosal resection without positive surgical margins. Median patient survival (IQR), after treatment, was 6 months (2.5–24.0).

## 4 Discussion

Renal cell carcinoma is the most common solid lesion within the kidney and accounts approximately 90 % of all kidney malignancies [1]. It comprises different RCC subtypes with specific histopathological characteristics [42]. Clear cell RCC is the most common histopathological entities of RCC [40] and clear cell RCC has worse prognosis compared to other histopathological entities [43,44], even after stratification for stage and grade [45].

Indeed, clear cell RCC has an abundant blood supply and can metastasize to several organs [46]. Metastasis routes include hematogenous, lymphogenous, renal capsule, renal pelvis and ureteric routes [37], which explains the wide variety of organs that can be RCC metastatic sites.

Currently, EAU guidelines [40] and French ccAFU (comité de cancérologie de l’Association Française d’Urologie) guidelines [47] on management of kidney cancer recommends using MSKCC and IMDB prognostic models to establish the prognosis of patients with metastatic RCC, in order to select the appropriate therapy. Thus, EAU [40] and French ccAFU guidelines [47] recommend offering surgical treatment, when technically feasible, of metastases in patients with oligo-metastatic RCC, in order to defer the initiation of systemic treatment.

Our systematic review suggests that solitary gastric metastasis of RCC are scarce. Herein, we report the 12th case of solitary gastric metastasis, discovered 6 years after local treatment of RCC. For the 11th cases reported in the literature, the median age of the patients was 68.0 years, of whom 82 % were men. The most frequent locations of metastasis were the middle body of the stomach (45 %), followed by the upper body of the stomach (36 %) and the lower body of the stomach (18 %). The median time between diagnosis of RCC and metachronous gastric metastasis was 8.5 years. A surgical treatment of the metastasis was performed in 64 % of the patients and an endoscopic treatment in 27 % of the cases. Thus, in case of solitary gastric metastasis of RCC, endoscopic or surgical treatment of the metastasis, allows to delay the initiation of systemic treatment, with median patient survival of 9 months after treatment. Our case has a similar age. The location of the metastasis was the middle body of the stomach. The median time between diagnosis of RCC and metachronous gastric metastasis was 6 years, lower than median time reported by the 11th cases. Two years after gastric metastasis surgical treatment, a hepatic hilum adenopathy recurrence occurred, treated by surgery and our median patient survival was higher (26 months versus 9 months).

However, in case of multiple metastatic sites, the stomach is a frequent localization of metastases. Thus, at gastric metastases diagnosis, 80 % of patients presented multiple metastatic sites, mainly lungs and bones. The median time between RCC diagnosis and metachronous gastric metastasis diagnosis was shorter in case of multiple metastatic sites (5.0 years versus 8.5 years in case of solitary gastric metastasis).

Surgical treatment, when it is feasible remains the best therapeutic option for a solitary gastric metastasis, resulting in significant survival prolongation in eligible patients. Indeed, median patient survival was higher after surgical treatment of gastric metastasis (9 months (3.4–15.8) versus 6 months (2.5–24.0) in case of solitary and multiple metastatic sites).

However, after surgical treatment of solitary gastric metastases, the risk of metastatic recurrence is significant. Among the 11 cases of solitary gastric metastasis reported in the literature, 3 patients presented a metastasis recurrence. Our case presented a hepatic hilum adenopathy recurrence, treated by surgery, two years after gastric metastasis surgical treatment.

## 5 Conclusion

Our systematic review suggests that solitary gastric metastasis of RCC are scarce. In comparison of patients with multiple metastatic sites, the median survival of patients with solitary gastric metastasis is longer. When it is feasible, surgical or endoscopic treatment of the solitary gastric metastasis should be performed, whenever the patient is fit for surgery, in order to delay the initiation of systemic treatment. Nevertheless, the risk of metastatic recurrence is significant and must be taken into consideration in the therapeutic strategy.

## Declaration of Competing Interest

None.

## Funding

None.

## Ethical approval

According to French legislation, retrospective studies are not subject to an ethics committee.

## Consent

Written informed consent was obtained from the patient for publication of this case report and accompanying images. A copy of the written consent is available for review by the Editor-in-Chief of this journal on request.

## Author’s contribution

Dr. Thomas Prudhomme: Data collection, Data analysis, Manuscript writing

Dr. Charlotte Maulat: Data collection, Data analysis

Dr. Guillaume Péré: Data collection, Data analysis

Dr. Fatima-Zohra Mokrane: Data collection, Data analysis

Pr. Michel Soulié: Data collection, Data analysis

Pr. Fabrice Muscari: Data collection, Data analysis, Manuscript writing.

## Registration of research studies

Not applicable.

## Guarantor

Thomas Prudhomme.

## Provenance and peer review

Not commissioned, externally peer-reviewed.
