# Supplementary material for: Are gastric metastases of renal cell carcinoma really rare? A case report and systematic review of the literature
Source: Int J Surg Case Rep. 2021 Apr 6;82:105867. doi: 10.1016/j.ijscr.2021.105867 (PMC8055614; doi:10.1016/j.ijscr.2021.105867)
Supplement: Supplementary file 1 [file mmc1.docx]

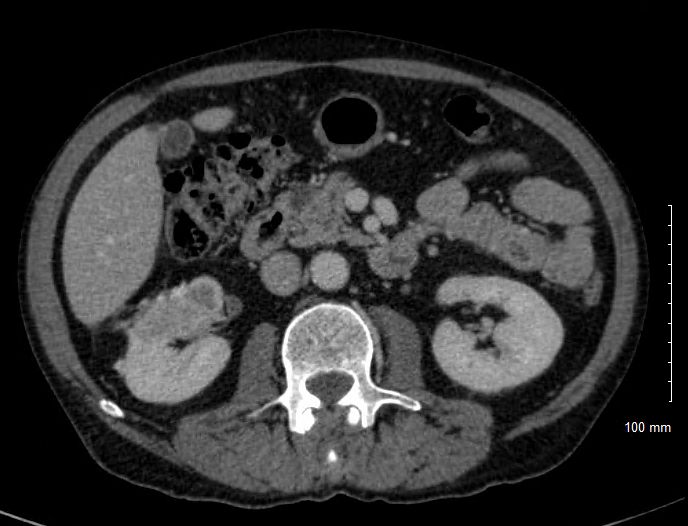


**Supplemental Figure 1.** Homolateral RCC recurrence 15 months after right open partial nephrectomy
