# Supplementary figures and images for: Are gastric metastases of renal cell carcinoma really rare? A case report and systematic review of the literature
Source: Int J Surg Case Rep. 2021 Apr 6;82:105867. doi: 10.1016/j.ijscr.2021.105867 (PMC8055614; doi:10.1016/j.ijscr.2021.105867)

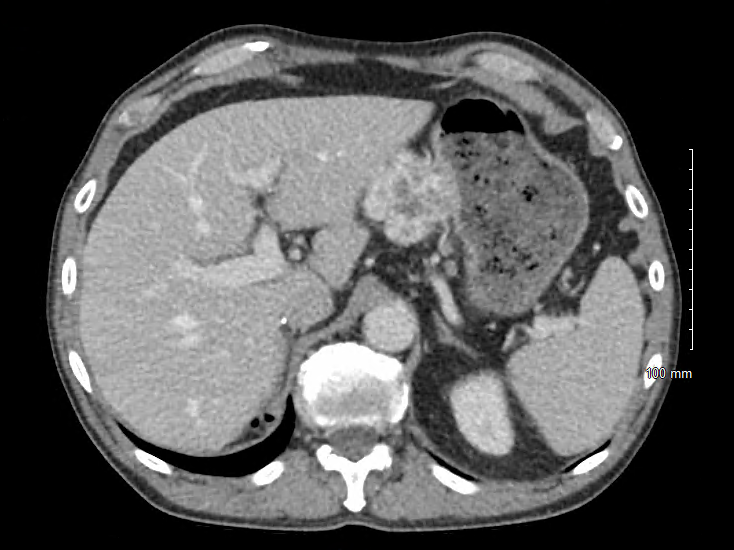


**Supplemental Figure 2.** Lesser curvature gastric metastases at CT scan

Supplement: Supplementary file 2 [file mmc2.docx]
